# How to Get All Trials Reported: Audit, Better Data, and Individual Accountability

**DOI:** 10.1371/journal.pmed.1001821

**Published:** 2015-04-14

**Authors:** Ben Goldacre

**Affiliations:** 1 Senior Clinical Research Fellow, Centre for Evidence-Based Medicine, Department of Primary Care Health Sciences, University of Oxford, Oxford, United Kingdom; 2 Research Fellow, Department of Non-communicable Disease Epidemiology, London School of Hygiene & Tropical Medicine, London, United Kingdom

## Abstract

Reflecting on the new WHO statement on clinical trial registration and reporting, Ben Goldacre proposes simple solutions for ensuring clinical trial results are made publicly available.

In this week’s *PLOS Medicine*, the World Health Organization (WHO) publishes a landmark position statement, requiring all trials to make their methods and results available [1]. This represents important progress on a long-standing and global structural problem that has a clear, negative impact on patient care. The best currently available evidence shows that the methods and results of clinical trials are routinely withheld from doctors, researchers, and patients [2–5], undermining our best efforts at informed decision making. From this point forward, whenever the methods and results of a trial are withheld, doctors, patients, researchers, campaigners, and health care providers will be able to point at an unambiguous statement from WHO.

Delivering definitive change, however, will require more than positive statements and good intentions. The first quantitative data demonstrating publication bias in clinical trials—and clear call for trial registries—was published in 1986 [6]. Anyone withholding the methods and results of a clinical trial is already in breach of multiple codes and regulations, including the Declaration of Helsinki, various promises from industry and professional bodies, and, in many cases, the United States Food and Drug Administration (FDA) Amendment Act of 2007. Indeed, a recently published cohort study of trials in clinicaltrials.gov found that more than half had failed to post results; and even though the FDA is entitled to issue fines of $10,000 a day for transgressions, no such fines have ever been levied [3].

In the face of such slow progress, this commentary sets out some practical suggestions for auditing, performance tables, accountability, codes of conduct, and better data that should help to drive up standards and prevent trial reports being withheld from those who need them most.

## What Should Trials Transparency Look Like, and How Do We Achieve It?

The WHO statement calls for summary results to be both posted on a registry and submitted to a journal within 12 months. However, it is worth noting that academic journal publication may ultimately prove to be a red herring, as an indicator of transparency. Academic publishing decisions can be arbitrary, and introduce lengthy delays in access to knowledge. Furthermore, there is a growing body of evidence demonstrating that journals often fall short of the basic expected standards for reporting of clinical trials. It is commonplace to find that primary outcomes have been switched, for example [7]; findings are routinely “spun” [8]; and compliance with reporting standards such as CONSORT is highly variable. When compared with the long and formal structured Clinical Study Reports created for all industry-sponsored trials, academic papers have been shown to be incomplete and inconsistent [9].

However, since all clinical trials are fundamentally similar—when compared, for example, with the myriad study designs in molecular biology—it has been possible to develop reporting standards and operationalise these. Reporting results onto a structured database, such as the results tab of clinicaltrials.gov [10], has many preferable features: there is minimal delay, there is compulsory reporting of features that are required; and there is no possibility to switch pre-specified outcomes or other forms of reporting misconduct. Put simply, there is a box to report the pre-specified primary outcome, and it has to be filled. Recent research has shown that academic journal reports are inconsistent with those on clinicaltrials.gov [2] and contain less complete information on methods, results, and adverse events [11]. Furthermore, International Committee of Medical Journal Editors (ICMJE) member journals have explicitly stated that they will not reject trial reports on the grounds that the results have already appeared on clinicaltrials.gov, and that they do not regard registry results reporting as prior publication [12]. Lastly, clinicaltrials.gov is clear that they will accept results on any trial, from any era, on any treatment, from any territory. This negates a key defence commonly cited by trialists and sponsors when facing calls for greater transparency: that journals reject “negative” results. All trials can now be reported, immediately, using clinicaltrials.gov as a first or last resort, if the trialist is willing. The question remains: how can we ensure this is done?

## The Need for Audit

One key element is likely to lie in medicine’s most basic research tool. Audits are routinely conducted on local service issues, such as infection rates, or waiting times, but rarely on broader structural issues such as publication bias, even though the impact of the latter on patient care is likely to be greater and global. Indeed, it is peculiar that for many years trial registration was considered an end in itself, when in reality registration is only of value as the raw material for publication audit.

The basic structure for a routine ongoing audit of results reporting is simple: using a register, identify trials that completed more than 12 months ago; establish, through whatever means, whether results from the trial have been reported; and post the date of results appearing to the register. From this, it is trivial to derive performance metrics for individual companies, funders, drugs, disease areas, institutions, and investigators.

This is highly specific and accountable information that can be used for practical good. Firstly, the very act of creating such data would allow us to name and shame poor performers, and also to reward best practice. Furthermore, those falling behind can identify and learn from those who are successfully meeting their obligations to patients.

The results of the audit can also be used to inform medical decision making. While it is unwise for doctors to use their prescription pads to pursue political goals, transparency metrics for an individual drug company are valuable context for interpreting data on the benefits of their products. For example, suppose there are two treatments of apparently equal benefit in meta-analysis, but one is made by a company with a proven track record of complete transparency, with 95% of all information available, while the other is made by a company with clear record of withholding information. The clinically cautious approach is to prescribe the treatment for which the results are more reliable, from the company that is more transparent.

Audit data can also be used by ethics committees and institutional review boards (IRBs). Withholding the results of clinical trials is unethical and harms patients. Those guilty of such misconduct could be banned from conducting further trials on patients until their previous trials have been made available. Indeed, even in the absence of such audit data, it would be trivial for all IRBs to ask one simple question of all those applying to conduct a trial: “Have you been involved in any clinical trial, which completed more than 12 months ago, for which the results remain inaccessible?”

Professional bodies and professional regulators, similarly, can now incorporate the WHO guidance into their codes of conduct and create mechanisms to ensure it is acted upon, for example by opening formal investigations when contacted over concerns around results being withheld by individual researchers or clinicians, and triggering disciplinary action whenever audit shows that the codes have been broken. It is rare, in professional regulation, to have data on transgressions created so rapidly and so unambiguously; it would be wrong to neglect this opportunity to improve standards. Patient groups, lastly, could write open letters to all companies and researchers withholding methods and results of trials on treatments taken by their members, represent their constituencies by holding individuals to reasonable account, and again help improve compliance.

## The Practicalities of Audit

Such audit can be conducted locally, centrally, or ideally, both. Since the recent rejuvenation in policy discussion in the United Kingdom on withheld trials, there have been small local audits conducted by various bodies, including sections of the Health Research Authority (as yet unpublished); the National Institute of Health Research (as yet unpublished); the Medical Research Council (to produce an estimate of publication bias for a 2012 UK parliamentary inquiry into trials transparency [13], but as yet unpublished); and an ongoing audit, on which I am a collaborator, covering trials in the University of Oxford. For the latter, alongside our findings, we also plan to publish our practical experiences of conducting the audit, with a boilerplate protocol that can be used by others in order to help make local audits simpler and produce comparable data. Such audits could and should be conducted and published routinely by all government research funders, industry sponsors, and institutions, to help ensure that all trials are reported.

Central audit is also desirable, and can be readily worked into existing trial registry workflows. At present, a completed trial without an associated results report on a registry may represent a transgression, but it may also represent an administrative failure. Publishing performance data and acting upon it will incentivise trialists to update their records. Worse still, it is currently impossible to establish on clinicaltrials.gov whether a completed trial has successfully requested an extension for reporting (whatever one might think of such exemptions), because this information is not posted; if data fields on such exceptions are routinely and transparently posted in public onto the database, compliance and transparency rankings can be automatically generated at no cost.

When discussing efficiencies, it is important to be clear, however, that the cost of even manual audit is trivial in comparison to the cost of conducting a randomised trial. Producing accessible knowledge for clinical decision making is the key purpose of a trial. Once a trial has been conducted—at great cost—and left unreported, then the small and final marginal cost of making its results available represents better value for money than almost any other step in the research process.

## What to Do about Past Trials

The emphasis by WHO on having access to all trials, from the past as well as the future, is particularly important and welcome. It is clinically highly relevant because the overwhelming majority of prescriptions today are for treatments that came onto the market—and were therefore researched—over the preceding decades rather than the past five years. The question, however, is how to prioritise access to such information, since there is no sense in resources being deployed on sharing evidence that is no longer relevant to current practice. There are many options. One is to proactively release information, prioritising by some metric of clinical need, such as the number of patients affected; or usage, such as the number of prescriptions issued for that class of treatments; or even a complex model built around power calculations and the likelihood of the withheld data changing the conclusions of the best current systematic review.

A simpler option, however, is for thorough retrospective registration of clinical trials to act as a “menu” from which doctors, researchers, and patients can request further disclosure of full methods and results, with appropriate transparency around the request and adjudication process. This is an attractive option since registration is low cost, but it does present one previously undocumented challenge. Through the AllTrials.net campaign, we are currently conducting an audit of companies’ policies on trials transparency, to create a Trials Transparency Index. In doing so, we have met a large number of individual companies to ask about gaps in their policies. One recurring theme, on the issue of retrospective registration, is that registries often require detailed administrative information (such as an IRB approval number) that is not readily traceable 20 years after a trial was completed. It may therefore be pragmatic to take a more permissive approach to completeness of certain data fields, with missing items replaced by an explanatory note where absolutely necessary, in preference to a trial not being retrospectively registered at all.

## Conclusion

The position statement from WHO is powerful and welcome, but previous calls for registration were not enough to fix publication bias, and positive statements require practical implementation. The solution is likely to lie in simple audit, providing better data for individual accountability. This can be delivered at low cost through a routine audit cycle to identify completed but unreported trials on all registries, with public performance tables that will incentivise trialists to ensure their registry entries reflect their compliance. Local audit will facilitate data-checking and ensure local accountability. As with all audit cycles throughout clinical practice this data must be acted on, with those who are guilty of research misconduct in withheld trials exposed to public scrutiny and local performance management; investigations automatically triggered by their professional regulators; and denied access to further trial participants. Lastly, doctors and patients can act on withheld data exposed by audit and consider avoiding treatments—or indeed whole companies—where there is clear evidence that the data on those interventions is comparatively unreliable.

These are simple processes that should have been integrated into the information ecosystem of evidence-based medicine from the outset. We cannot make truly informed decisions when vitally important information on the methods and results of clinical trials is routinely withheld, and yet we have tolerated this simple, fixable, pervasive flaw in evidence-based medicine for many decades. The doctors and patients of the future may well look back on this phenomenon with amazement, much as we look back on mediaeval bloodletting.
